# The PrFRS2-PrMYB75a module regulates petal coloration in flare tree peony (*Paeonia rockii*)

**DOI:** 10.1093/hr/uhaf288

**Published:** 2025-10-16

**Authors:** Fang-Ting Qi, Jia-Ning Han, Fang-Yun Cheng, Yuan Zhong, Lei Zhang, Yi-Fan Zhao, Xiao-Fang Liu

**Affiliations:** State Key Laboratory of Efficient Production of Forest Resources, Beijing Forestry University, Beijing 100083, China; National Engineering Research Center for Floriculture, Beijing Forestry University, Beijing 100083, China; Peony International Institute, Beijing Forestry University, Beijing 100083, China; School of Landscape Architecture, Beijing Forestry University, Beijing 100083, China; State Key Laboratory of Efficient Production of Forest Resources, Beijing Forestry University, Beijing 100083, China; National Engineering Research Center for Floriculture, Beijing Forestry University, Beijing 100083, China; Peony International Institute, Beijing Forestry University, Beijing 100083, China; School of Landscape Architecture, Beijing Forestry University, Beijing 100083, China; State Key Laboratory of Efficient Production of Forest Resources, Beijing Forestry University, Beijing 100083, China; National Engineering Research Center for Floriculture, Beijing Forestry University, Beijing 100083, China; Peony International Institute, Beijing Forestry University, Beijing 100083, China; School of Landscape Architecture, Beijing Forestry University, Beijing 100083, China; State Key Laboratory of Efficient Production of Forest Resources, Beijing Forestry University, Beijing 100083, China; National Engineering Research Center for Floriculture, Beijing Forestry University, Beijing 100083, China; Peony International Institute, Beijing Forestry University, Beijing 100083, China; School of Landscape Architecture, Beijing Forestry University, Beijing 100083, China; State Key Laboratory of Efficient Production of Forest Resources, Beijing Forestry University, Beijing 100083, China; National Engineering Research Center for Floriculture, Beijing Forestry University, Beijing 100083, China; Peony International Institute, Beijing Forestry University, Beijing 100083, China; School of Landscape Architecture, Beijing Forestry University, Beijing 100083, China; State Key Laboratory of Efficient Production of Forest Resources, Beijing Forestry University, Beijing 100083, China; National Engineering Research Center for Floriculture, Beijing Forestry University, Beijing 100083, China; Peony International Institute, Beijing Forestry University, Beijing 100083, China; School of Landscape Architecture, Beijing Forestry University, Beijing 100083, China; State Key Laboratory of Efficient Production of Forest Resources, Beijing Forestry University, Beijing 100083, China; National Engineering Research Center for Floriculture, Beijing Forestry University, Beijing 100083, China; Peony International Institute, Beijing Forestry University, Beijing 100083, China; School of Landscape Architecture, Beijing Forestry University, Beijing 100083, China

## Abstract

Flower color is an essential biological and ornamental trait in plants. *Paeonia rockii* (flare tree peony, FTP) exhibits diverse flower colors, characterized by a distinctive basal flare in petals, which enhances its ornamental and ecological value. However, while previous research has mainly focused on flare formation, the regulatory mechanisms controlling the background color of petals remain unclear. This study identifies a novel regulatory module governing petal background coloration in FTP. Within this module, PrMYB75a acts as the central regulator to promote anthocyanin accumulation, as evidenced by stable transformation in *Arabidopsis thaliana* and tobacco (*Nicotiana tabacum*), as well as virus-induced gene silencing in FTP. Furthermore, yeast one-hybrid, dual-luciferase reporter, and electrophoretic mobility shift assays collectively demonstrated that PrMYB75a directly activates two key anthocyanin structural genes, *PrCHS1* and *PrANS*, by interacting with MYB-binding sites nearest to the *ATG* start codon in their promoters. Additionally, we identified an upstream regulator, PrFRS2, which activates both *PrMYB75a* and *PrANS* by binding to the FAR1/FHY3-binding sites in their promoters. Modulation of *PrFRS2* expression levels through gene silencing and overexpression resulted in alterations in flower pigmentation in both FTP and tobacco. In summary, within the PrFRS2-PrMYB75a module, PrFRS2 indirectly activates *PrCHS1* and *PrANS* by regulating *PrMYB75a*, or directly activates *PrANS*, leading to anthocyanin accumulation in FTP purple petals. This module represents a novel regulatory mechanism of petal background coloration in FTP, providing new perspectives on color variation in flowering plants and offering genetic resources for the improvement of the flower color trait in tree peonies.

## Introduction

Flower color, as a key phenotypic trait, mediates critical biological interactions and carries significant aesthetic value [1–3]. The tree peony (*Paeonia* sect. *Moutan*), renowned as the ‘King of Flowers’, has been cultivated in China for over 1400 years due to its visual appeal, medicinal properties, and emerging potential as an oilseed crop [4]. Currently, there are mainly two groups of cultivated tree peonies in gardens: common tree peony (CTP) (*Paeonia suffruticosa*) and flare tree peony (FTP) (*Paeonia rockii*) [5]. Notably, FTP exhibits vigorous growth, abundant flowers, and strong tolerance to stressful environmental conditions, including cold, drought, and poor soil, indicating its great potential for industrial applications [6]. While both display various flower colors, the distinctive colored flare at the base of the petals distinguishes FTP from CTP. Several studies on CTP have investigated petal color formation [7–9]. However, in FTP, research has mainly focused on the development of flare color [10–13], while the uniform background color covering most of the petal has received comparatively less attention. Thus, although certain investigations have examined the pigment components in FTP petals [14, 15], the molecular mechanisms governing petal background coloration remain poorly understood.

Anthocyanins are recognized as the principal pigments responsible for floral coloration in most angiosperms, among various influencing factors [16]. The coloration of tree peony blossoms is mainly attributed to three types of anthocyanins, including cyanidin (Cy), peonidin (Pn), and pelargonidin (Pg) derivatives [9, 14]. Structural genes and transcription factors (TFs) play important roles in the anthocyanin biosynthesis pathway (ABP) [17]. ABP-related structural genes can be divided into two groups: early biosynthesis genes (EBGs) and late biosynthesis genes (LBGs). EBGs include *phenylalanine ammonia-lyase* (*PAL*), *4-coumarate-CoA ligase* (*4CL*), *chalcone synthase* (*CHS*), *chalcone isomerase* (*CHI*), and *flavonoid 3-hydroxylase* (*F3H*), while LBGs include *dihydroflavonol 4-reductase* (*DFR*), *anthocyanidin synthase* (*ANS*), *glycosyltransferase* (*GT*), *acyltransferase* (*AT*), and *methyltransferase* (*MT*) [16, 18]. In FTP, structural genes such as *PrCHS*, *PrDFR*, and *PrANS* have been associated with anthocyanin accumulation, particularly in the dark basal flare of petals [11, 12, 19]. Similarly, the high expression levels of *PsCHS*, *PsDFR*, *PsF3H*, and *PsANS* significantly contributed to anthocyanin production in CTP petals [20–23]. The conservation of these anthocyanin structural genes across flared and nonflared tree peony groups suggests that upstream regulatory factors might play a more critical role in both petal background coloration and basal flare formation.

In most plants, MYB TFs are the main regulators of the anthocyanin structural genes, functioning either independently or as part of the MYB-bHLH-WD40 (MBW) complex [24]. Specifically, R2R3-MYB activators from subgroup 5 (SG5) and subgroup 6 (SG6) of the MYB family are crucial for anthocyanin biosynthesis in various species [25, 26]. Examples include FaMYB5 (SG5) in strawberry (*Fragaria* × *ananassa*), AtMYB75 (SG6) in *Arabidopsis* (*Arabidopsis thaliana*), and PyMYB10 (SG6) in pear (*Pyrus* spp.) [27–29]. SG5/6 R2R3-MYBs have also been reported to enhance anthocyanin production in tree peony [30–33]. In CTP, PsMYB57 (SG6) and PsMYB58 (SG6) increase anthocyanin levels in petals, but their functional mechanisms are not well understood [34, 35]. In FTP, several *R2R3-MYB* genes, including *PsMYB12* (SG5), *PrMYB5* (SG5), *PrMYBa2* (SG5), and *PrMYBa3* (SG6), have been identified as anthocyanin activators in basal flare by activating one or more target structural genes [11–13]. Nevertheless, the specific roles of these *PrMYBs* in the petal background coloration remain unreported. To date, relatively few MYBs associated with background coloration have been found in FTP, indicating a necessity for further investigation.

Beyond the core MBW complex, the regulatory network of anthocyanin biosynthesis in tree peony remains largely unexplored, although a few TFs, such as PsHY5, have been implicated [36]. In contrast, diverse TFs that upstream regulate *MYB*s or directly regulate ABP-related structural genes have been reported to influence anthocyanin accumulation in various plants [25, 37]. Notably, FAR1-RELATED SEQUENCE (FRS) TFs, which are essential for light signaling and plant growth [38, 39], have also been found to participate in anthocyanin biosynthesis [40–43]. FAR-RED ELONGATED HYPOCOTYL 3 (FHY3) and its homolog, FAR-RED-IMPAIRED RESPONSE 1 (FAR1), are the founding members of the FRS TF family [44]. They belong to the FRS subfamily 1 (SG1), which includes FRS1, FRS2, and FRS4. FHY3/FAR1 can regulate numerous genes related to plant growth and stress responses, exhibiting functional diversity [38]. Recent research has linked the transcript levels of FAR1 to flavonoid accumulation in chrysanthemum (*Chrysanthemum* × *morifolium*), *Camellia sasanqua*, and blood orange (*Citrus sinensis*) [42, 43, 45]. In grape (*Vitis vinifera*), VvFHY3 directly binds to FAR1/FHY3-binding sites (FBS) in the promoters of structural genes (*VvCHS1*, *VvF3H1*, and *VvLDOX*), thereby regulating anthocyanin synthesis [41]. Although previous studies have identified FRS TFs as novel regulators of anthocyanin, it remains unclear whether *FRS* genes are associated with anthocyanin biosynthesis in tree peonies.

The aim of our work is to investigate the molecular mechanisms by which MYB TFs regulate FTP petal background coloration, as well as potential upstream regulators in this process, such as FRS TFs. We used the transcriptome database of white and purple petals (excluding the basal flare) to identify a differentially expressed *R2R3-MYB* gene, designated *PrMYB75a*. Further analysis demonstrated that PrMYB75a activates the expression of *PrCHS1* and *PrANS* by binding to their promoters, resulting in increased anthocyanin levels and the formation of purple petals. Moreover, we located an FRS TF, PrFRS2, that functions upstream of *PrMYB75a*, contributing to anthocyanin accumulation and petal background coloration by activating both *PrMYB75a* and *PrANS*. This study is the first to identify an FRS TF associated with flower color in tree peonies and to clarify the regulatory mechanism of the PrFRS2-PrMYB75a module in FTP petal background coloration. These findings provide valuable insights into the hierarchical regulatory network governing plant anthocyanin metabolism and the formation of complex flower coloration patterns.

## Results

### Anthocyanins are primarily responsible for the background coloration of purple petals in FTP.

**Figure 1:**
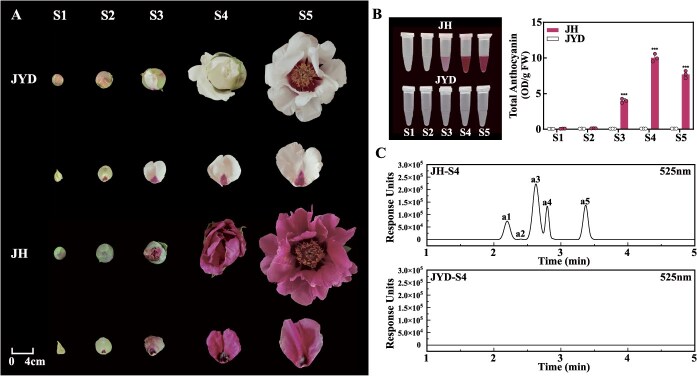
Identification and quantification of pigments in JH and JYD petals. (A) Phenotypes of JH and JYD flowers at five developmental stages. S1: green petals without a basal flare; S2: green petals with a colored basal flare; S3: slightly colored petals with a basal flare; S4: buds beginning to bloom; S5: fully bloomed flowers. (B) Total anthocyanin extraction and content analysis in JH and JYD petals (excluding the basal flare) at five stages. Data represent the mean ± standard deviation (SD) of three biological replicates, with asterisks indicating significant differences determined by a two-sided Student’s *t*-test (^***^P < 0.001). (C) HPLC chromatograms of anthocyanins extracted from JH petals at S4, measured at 525 nm. Peak a1: cyanidin 3,5-di-*O*-glucoside (Cy3G5G); peak a2: pelargonidin 3,5-di-*O*-glucoside (Pg3G5G); peak a3: Pn3G5G; peak a4: cyanidin 3-*O*-glucoside (Cy3G); peak a5: peonidin 3-*O*-glucoside (Pn3G). Peak identification and mass spectrum data are provided in Table S1.

To clarify the key factors determining petal background coloration in FTP, we selected two cultivars: the purple-flowered ‘Jing Hong’ (JH) and the white-flowered ‘Jing Yu Dan’ (JYD). Both cultivars feature a basal flare in their petals but exhibit contrasting background colors. During the five developmental stages (S1–S5), both cultivars had light green petals at S1 and S2. In contrast, from S3 to S5, JH gradually developed purple petals, whereas JYD remained white (Fig. 1A). Analysis of total anthocyanin content in the petals (excluding the basal flare) revealed that the anthocyanin level of JH was low at S1 and S2, then increased significantly at S3, peaked at S4, and declined slightly at S5 (Fig. 1B). In contrast, JYD petals (excluding the basal flare) exhibited negligible anthocyanin accumulation at all stages (Fig. 1B). The anthocyanin components of the petals (excluding the basal flare) at S4 were then detected using high-performance liquid chromatography-ion trap mass spectrometry (HPLC-MS) (Table S1). HPLC analysis revealed that JH contains two cyanidin derivatives (peaks a1 and a4), two peonidin derivatives (peaks a3 and a5), and one pelargonidin derivative (peak a2), while JYD contains no anthocyanins (Fig. 1C). Based on these data, we conclude that anthocyanins are the fundamental determinants of background coloration in FTP purple petals, with peonidin 3,5-di-*O*-glucoside (Pn3G5G) serving as the predominant pigment (Fig. S1), and S3 marking the onset of anthocyanin production.

### *PrMYB75a* is involved in anthocyanin biosynthesis in purple petals

**Figure 2:**
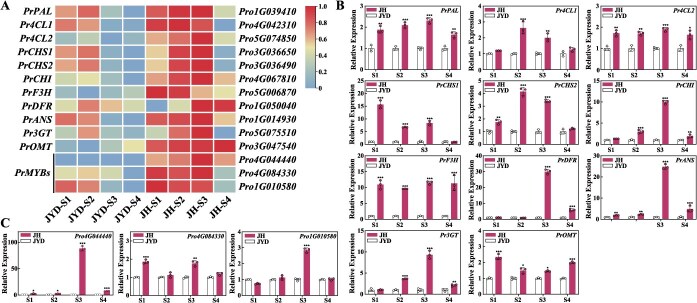
Expression analysis of candidate genes related to anthocyanin biosynthesis in JH and JYD petals. (A) The heatmap displays the transcripts per million (TPM) values of 11 ABP-related structural genes and three *PrMYB*s in JH and JYD petals (excluding the basal flare) at S1–S4. (B, C) Relative expression levels of 11 structural genes (B) and three *PrMYBs* (C) in JH and JYD petals (excluding the basal flare) at S1–S4 by RT-qPCR assay. Data represent the mean ± standard deviation (SD) of three biological replicates, with asterisks indicating significant differences determined by a two-sided Student’s *t*-test (^*^P < 0.05, ^**^P < 0.01, ^***^P < 0.001).

Given the established role of anthocyanin accumulation in the background coloration of FTP petals, we screened candidate ABP-related genes by comparing the transcriptome data from JH and JYD petals (excluding the basal flare) at S1–S4. Fourteen differentially expressed genes (DEGs) were identified as upregulated in JH petals (Fig. 2A, Table S2). According to the NCBI database, these DGEs were classified into seven EBGs (*PrPAL*, *Pr4CL1*, *Pr4CL2*, *PrCHS1*, *PrCHS2*, *PrCHI*, and *PrF3H*), four LBGs (*PrDFR*, *PrANS*, *Pr3GT*, and *PrOMT*), and three potential *PrMYB* genes (*Pro4G044440*, *Pro4G084330*, and *Pro1G010580*) (Table S3). Reverse transcription-quantitative real-time PCR (RT-qPCR) analysis confirmed significantly higher expression of seven EBGs in JH at S1–S3 and four LBGs at S3–S4, compared to JYD (Fig. 2B). Furthermore, all three *PrMYB*s exhibited considerably increased transcript levels in JH petals (excluding the basal flare), with *Pro4G044440* displaying more than a 100-fold increase at S3 (Fig. 2C). These expression profiles suggest that the identified DEGs, especially the *PrMYBs*, may be involved in anthocyanin synthesis in purple FTP petals.

**Figure 3:**
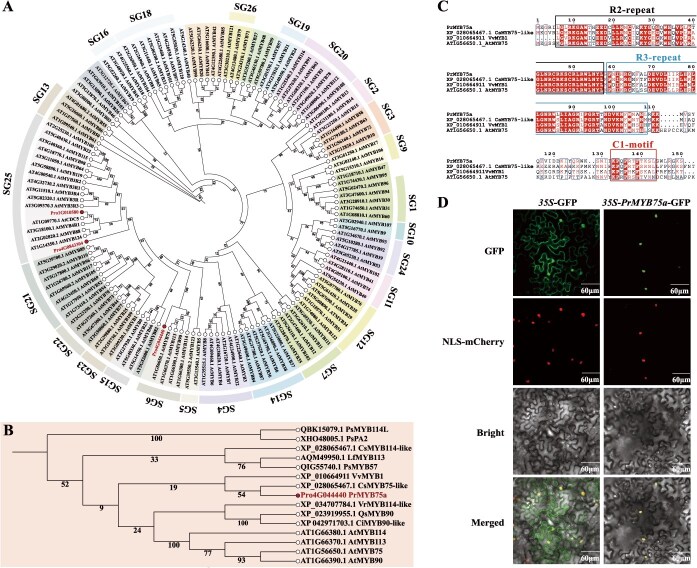
Bioinformatic analysis and subcellular localization of PrMYB75a. (A) Phylogenetic analysis of three potential PrMYBs and 131 *Arabidopsis* MYB TFs. (B) Phylogenetic analysis of PrMYB75a and 14 SG6 MYB TFs. These two neighbor-joining trees were generated using MEGA4 with the p-distance model and 1000 bootstrap replicates. The accession numbers of TFs are provided in Tables S4 and S5. (C) Multiple sequence alignment and motif analysis of PrMYB75a with three R2R3-MYB TFs: CsMYB75a-like (XP_028065467.1), VvMYB1 (XP_010664911), and AtMYB75 (AT1G56650.1). The conserved R2 repeat, R3 repeat, and C1 motif are indicated by rectangles. (D) Subcellular localization of PrMYB75a in the leaf epidermal cells of *N. benthamiana*. The nuclear marker mCherry protein was co-infiltrated with the *35S*-GFP (control) and *35S*-*PrMYB75a*-GFP vectors. GFP: GFP channel; NLS-mCherry: nuclear localization signal; Bright: light microscopy image; Merged: the merged image of GFP, NLS-mCherry, and Bright channels.

To predict the functions of potential *PrMYBs*, we conducted a phylogenetic analysis using their amino acid sequences with 131 *Arabidopsis* MYB proteins (Table S4). This analysis revealed that Pro4G044440 clustered with SG6 MYB TFs, while Pro4G084330 and Pro1G010580 clustered with SG25 MYB TFs (Fig. 3A). Since anthocyanin activators typically cluster in the SG5 and SG6 clades [25], we selected Pro4G044440 (SG6) for further investigation. A subsequent phylogenetic tree containing 14 SG6 MYBs (Table S5) revealed that Pro4G044440 is homologous to CsMYB75-like (Fig. 3B), an acknowledged anthocyanin activator in purple tea (*C. sinensis*) [46], leading us to designate it as PrMYB75a. Multiple sequence alignment confirmed that PrMYB75a is an R2R3-MYB protein, with conserved R2 and R3 repeats at the N-terminus and a C1 motif at the C-terminus (Fig. 3C). Subcellular localization further verified that the PrMYB75a protein is localized in the nucleus (Fig. 3D). Collectively, these findings suggest a potential role for *PrMYB75a* in anthocyanin biosynthesis underlying the background coloration of FTP purple petals.

### Functional verification of *PrMYB75a* in the background coloration of purple petals

**Figure 4:**
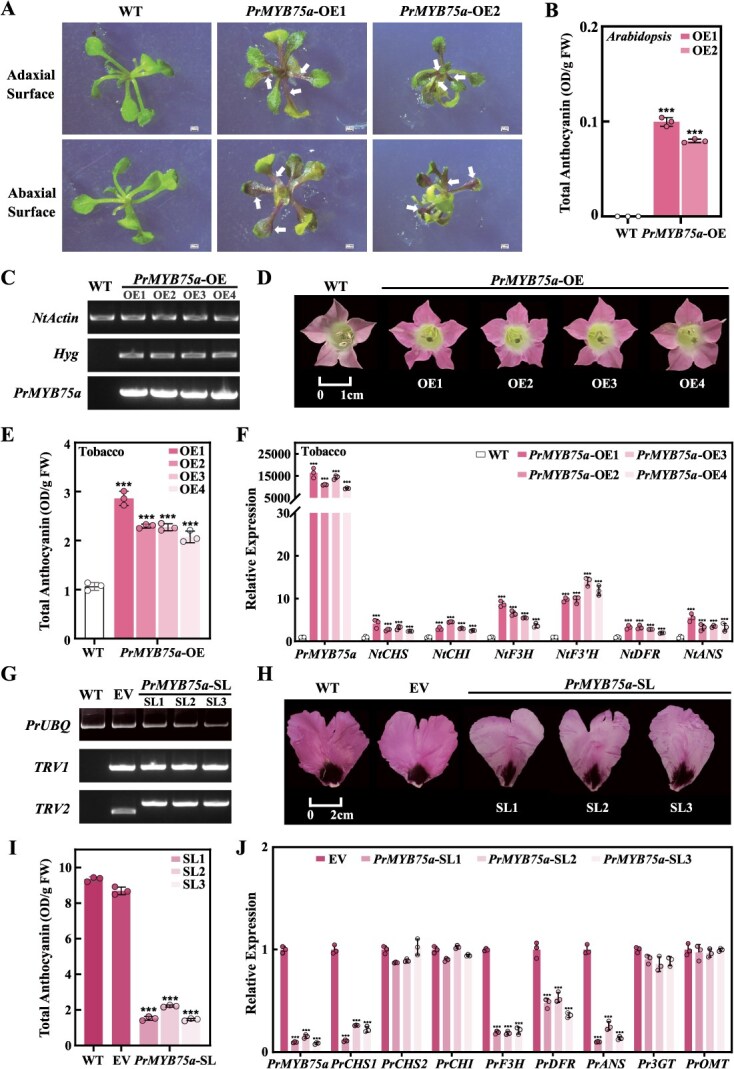
Functional verification of *PrMYB75a* in *Arabidopsis*, tobacco, and JH petals. (A) Phenotypes of the WT and two *PrMYB75a*-OE *Arabidopsis* lines observed under a stereomicroscope*.* The arrow indicates the purple regions. Scale bar: 0.5 mm. (B) Total anthocyanin content of WT and *PrMYB75a*-OE *Arabidopsis* lines. (C) PCR-positive verification of WT and four *PrMYB75a*-OE tobacco lines. *NtActin*: internal reference gene; *Hyg*: hygromycin resistance gene. (D) Flower phenotypes of WT and four *PrMYB75a*-OE tobacco lines. (E) Total anthocyanin content of WT and *PrMYB75a*-OE tobacco lines. (F) Relative expression levels of *PrMYB75a* and anthocyanin biosynthesis genes (*NtCHS*, *NtCHI*, *NtF3H*, *NtF3'H*, *NtDFR*, and *NtANS*) in WT and four *PrMYB75a*-OE tobacco lines by RT-qPCR assay. (G) Semiquantitative PCR (SQ-PCR) verification of target genes in WT, EV, and three *PrMYB75a*-SL petals. *PrUBQ*: internal reference gene; *TRV1*/*TRV2*: specific fragments in the TRV1/TRV2 vector. (H) Phenotype observation of WT, EV, and three *PrMYB75a*-SL petals. (I) Total anthocyanin levels in WT, EV, and *PrMYB75a*-SL petals. (J) Relative expression levels of *PrMYB75a* and anthocyanin structural genes (*PrCHS1*, *PrCHS2*, *PrCHI*, *PrF3H*, *PrDFR*, *PrANS*, *Pr3GT*, and *PrOMT*) in EV and *PrMYB75a*-SL petals by RT-qPCR assay. Data represent the mean ± SD of three biological replicates, with asterisks indicating significant differences determined by a two-sided Student’s *t*-test (***P < 0.001).

To determine whether *PrMYB75a* induces anthocyanin production, we successfully generated stable transgenic *Arabidopsis* and tobacco (*Nicotiana tabacum*) positive lines (Figs S2A and 4C), which exhibited obvious changes in pigmentation phenotypes. The rosette leaves and stems of two *PrMYB75a-*overexpressing (*PrMYB75a-*OE) *Arabidopsis* lines changed from green to purple (Fig. 4A), and the corollas of four *PrMYB75a-*OE tobacco lines shifted from pale pink to red (Fig. 4D). Consistent with these observations, all transgenic lines accumulated significantly more anthocyanin than wild-type (WT) plants (Fig. 4B and E). Furthermore, all *PrMYB75a*-OE *Arabidopsis* and tobacco lines showed upregulated expression of *PrMYB75* and anthocyanin pathway genes (*CHS*, *CHI*, *F3H*, *F3'H*, *DFR*, and *ANS/LDOX*) compared to WT (Fig. S2B and Fig. 4F). The above evidence indicated that *PrMYB75a* acts as an activator of anthocyanin biosynthesis.

Due to the limited genetic transformation methods available for tree peony, we transiently silenced *PrMYB75a* in JH buds using the TRV-mediated virus-induced gene silencing (VIGS) system to clarify its function in FTP. We established three experimental groups: *PrMYB75a-*silenced lines (*PrMYB75a*-SL, buds infiltrated with pTRV1 and *PrMYB75a*-pTRV2), empty vector lines (EV, buds infiltrated with pTRV1 and empty pTRV2), and untreated wild-type lines (WT) (Fig. 4G). The EV line was set as the control because it represented a similar phenotype and anthocyanin content with WT. All *PrMYB75a*-SL exhibited significantly faded petal background coloration and over 80% less anthocyanin content than the EV controls (Fig. 4H and I). Gene expression analysis revealed that the silencing efficiency of *PrMYB75a* exceeded 84% in all three silenced lines, accompanied by significantly reduced transcript levels of four ABP-related structural genes (*PrCHS1*, *PrF3H*, *PrDFR*, and *PrANS*) (Fig. 4J). Therefore, *PrMYB75a* is demonstrated to promote anthocyanin synthesis in the background coloration of FTP purple petals.

### PrMYB75a positively regulates anthocyanin biosynthesis by activating *PrCHS1* and *PrANS.*

**Figure 5:**
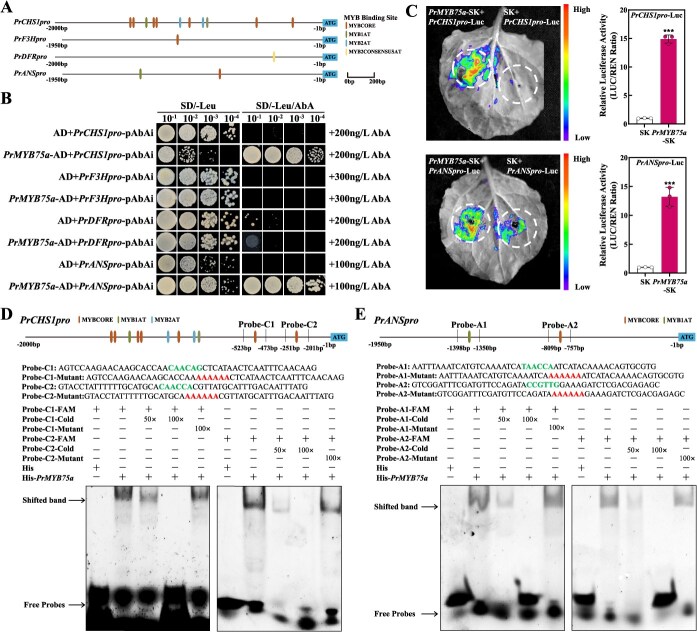
PrMYB75a activates *PrCHS1* and *PrANS* by binding to MBS motifs in their promoters. (A) Predicted MBS motifs in promoters of *PrCHS1*, *PrF3H*, *PrDFR*, and *PrANS*. Different MBS motifs (MYBCORE, MYB1AT, MYB2AT, and MYB2CONSENSUSAT) are highlighted with rectangles. (B) Y1H assay showed the interaction between PrMYB75a and four promoters (*PrCHS1pro*, *PrF3Hpro*, *PrDFRpro*, and *PrANSpro*). Y1H Gold cells containing *PrMYB75a*-AD and promoter-pAbAi constructs were cultured on SD/−Leu medium supplemented with Aureobasidin A (AbA). The AD + promoter-pAbAi combinations served as the control. (C) Dual-luc reporter assays demonstrated that PrMYB75a activates the promoters of *PrCHS1* and *PrANS*. Effector *PrMYB75a*-SK and the reporter constructs (*PrCHS1pro*-Luc or *PrANSpro*-Luc) were co-infiltrated into *N. benthamiana* leaves. The SK + promoter-Luc combinations served as the control. Representative images show Luc luminescence in leaves (left); the LUC/REN ratio reflects relative luciferase activity (right), with the control set to 1. Data represent the mean ± SD of three biological replicates, with asterisks indicating significant differences determined by two-sided Student’s *t*-test (***P < 0.001). (D and E) EMSA confirmed direct binding of PrMYB75a to MBS motifs in the promoters of *PrCHS1* (D) and *PrANS* (E). Probe-C1 (−523 to −473 bp) and Probe-C2 (−251 to −201 bp) contain the MYBCORE motif from *PrCHS1pro*. Probe-A1 (−1398 to −1350 bp) and Probe-A2 (−809 to −757 bp) contain one MYB1AT and one MYBCORE motif from *PrANSpro*, respectively. −FAM: FAM-labeled probes; −Cold: unlabeled competitor probes; −Mutant: unlabeled mutant probes; His: negative control protein. His-*PrMYB75a*: PrMYB75a protein. + and – indicate the presence or absence of probes or proteins. 50× and 100× denote molar excesses of unlabeled probes.

In order to investigate the regulatory mechanisms of *PrMYB75a*, we selected four structural genes (*PrCHS1*, *PrF3H*, *PrDFR*, and *PrANS*) based on their significantly downregulated expression levels in the *PrMYB75a*-SL lines. Promoter sequences (*PrCHS1pro*, *PrF3Hpro*, *PrDFRpro*, and *PrANSpro*) were cloned from JH petals, and cis-element analysis identified multiple MYB-binding sites (MBS) in each (Fig. 5A). Yeast one-hybrid (Y1H) assays confirmed that PrMYB75a specifically binds to *PrCHS1pro* and *PrANSpro*, but not to *PrF3Hpro* or *PrDFRpro* (Fig. 5B). Furthermore, dual-luciferase (Dual-luc) reporter assays *in vivo* demonstrated that PrMYB75a significantly increased *luciferase* (*Luc*) expression driven by *PrCHS1pro* and *PrANSpro* compared to controls (Fig. 5C). These results indicate that PrMYB75a directly activates the transcription of *PrCHS1* and *PrANS* through binding to their promoters.

To further pinpoint the specific binding sites of PrMYB75a on *PrCHS1pro* and *PrANSpro*, we conducted the electrophoretic mobility shift assay (EMSA). Since *PrANSpro* contains 2 MBS and *PrCHS1pro* contains 11, we shortened the *PrCHS1pro* fragment to obtain *PrCHS1pro*-1 (−2000 to −525 bp, with nine MBS) and *PrCHS1pro*-*2* (−2000 to −1218 bp, with five MBS) (Fig. S3A). The Y1H and Dual-luc reporter assays showed that *PrMYB75a* neither binds to nor activates these shortened promoters (Fig. S3B and C). These findings indicate that the region from −525 to −1 bp is required for the interaction between PrMYB75a and *PrCHS1pro*. As a result, we chose two MYBCORE motifs in *PrCHS1pro* (−525 to −1 bp), as well as one MYBCORE and one MYB1AT motif in *PrANSpro* (−1950 to −1 bp), to use as probes in the EMSA. EMSA confirmed PrMYB75a could bind to these four 5(6)-carboxyfluorescein (FAM)-labeled probes from *PrCHS1pro* and *PrANSpro*. Their binding was competitively inhibited by unlabeled cold probes, but not by unlabeled mutated probes (Fig. 5D and E). Thus, PrMYB75a directly binds to specific regions in *PrCHS1pro* and *PrANSpro*, preferentially targeting the MYBCORE motifs nearest the *ATG* start codon in two promoters, as well as the MYB1AT motif in *PrANSpro*.

### The expression of *PrMYB75a* is regulated upstream by PrFRS2

**Figure 6:**
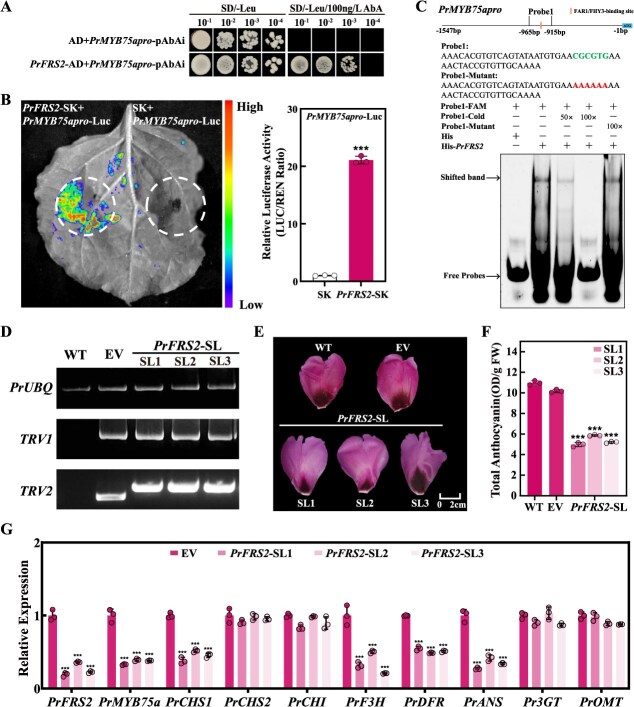
PrFRS2 activates *PrMYB75a* by binding to the FBS motif in its promoter. (A) Y1H assay showed PrFRS2 binding to the *PrMYB75* promoter (*PrMYB75apro*). Yeast cells containing *PrFRS2*-AD and *PrMYB75apro*-pAbAi vectors were grown on SD/−Leu with 100 ng/l AbA. The AD + *PrMYB75apro*-pAbAi combination served as the control. (B) Dual-luc reporter assay showed that PrFRS2 activates *PrMYB75apro. PrFRS2*-SK and *PrMYB75apro*-Luc were co-infiltrated into *N. benthamiana* leaves. The SK + *PrMYB75apro*-Luc combination was used as the control. Luc luminescence is represented in the image (left); relative luciferase activity was indicated by the LUC/REN ratio (right), with the control set to 1. (C) EMSA confirmed that PrFRS2 binds to the FBS motif in *PrMYB75apro*. Probe 1 (−965 to −915 bp) from *PrMYB75a* contains one FBS motif (CGCGTG). −FAM: FAM-labeled probes; −Cold: unlabeled competitor probes; −Mutant: unlabeled mutant probes; His: negative control protein. His-*PrFRS2*: PrFRS2 protein. + and – indicate the presence or absence of probes or proteins. 50× and 100× denote molar excesses of unlabeled probes. (D) SQ-PCR validated target genes in WT, EV, and three *PrFRS2*-SL petals. *PrUBQ*: internal reference gene; *TRV1*/*TRV2*: specific fragments in the TRV1/TRV2 vector. (E) Phenotypes of the WT, EV, and three *PrFRS2*-SL petals. (F) Total anthocyanin content of WT, EV, and three *PrFRS2*-SL petals. (G) Relative expression patterns of *PrFRS2*, *PrMYB75a*, and anthocyanin structural genes (*PrCHS1*, *PrCHS2*, *PrCHI*, *PrF3H*, *PrDFR*, *PrANS*, *Pr3GT*, and *PrOMT*) in EV and three *PrFRS2*-SL petals. Data represent the mean ± SD of three biological replicates, with asterisks indicating significant differences determined by a two-sided Student’s *t*-test (^***^P < 0.001).

To identify the upstream regulators of *PrMYB75a*, we isolated a 1547-bp *PrMYB75a* promoter (*PrMYB75apro*), which was identical in two cultivars (Fig. S4). Promoter analysis revealed multiple cis-elements within *PrMYB75apro*, suggesting the existence of several potential upstream regulators (Table S6). A Y1H screening experiment using *PrMYB75apro* as bait identified 69 unigenes through Sanger sequencing. Eleven of the 69 unigenes were classified into recognized TF families (Table S7), including PrFRS2, an FRS-family protein homologous to *Arabidopsis* AtFRS2 (Fig. S5A, Table S8). RT-qPCR assays indicated that *PrFRS2* was highly expressed in JH petals (excluding the basal flare) (Fig. S5B). Subcellular localization analysis revealed that PrFRS2 is a nuclear-localized protein (Fig. S5C). The Dual-luc reporter assay demonstrated that PrFRS2 significantly enhanced *Luc* expression driven by *PrMYB75apro*. Moreover, Y1H and EMSA further confirmed the direct binding of PrFRS2 to the FBS motif (CGCGTG) in *PrMYB75apro* (Fig. 6A–C). Finally, we determined that PrFRS2 directly binds to the FBS motif in the *PrMYB75a* promoter, thereby activating its transcription.

To validate the biological function of *PrFRS2* in anthocyanin accumulation in FTP petals, we silenced *PrFRS2* in JH buds using VIGS. This generated WT, EV, and three *PrFRS2*-silenced (*PrFRS2*-SL, buds infiltrated with pTRV1 and *PrFRS2*-pTRV2) lines (Fig. 6D). Compared to the EV controls, all three *PrFRS2*-SL petals exhibited faded background coloration and a reduction in anthocyanin concentration of more than 41% (Fig. 6E and F). Meanwhile, transcript levels of *PrFRS2* and *PrMYB75a* significantly decreased in these *PrFRS2*-SL petals. Furthermore, the expression of key ABP-related structural genes (*PrCHS1*, *PrF3H*, *PrDFR*, and *PrANS*) also reduced (Fig. 6G), similar to the gene expression profiles observed in *PrMYB75a*-SL petals. Therefore, we recognized PrFRS2 as an upstream transcriptional activator of *PrMYB75a*, which upregulates ABP-related structural genes to promote anthocyanin synthesis in FTP purple petals.

### PrFRS2 directly activates *PrANS* to enhance anthocyanin production

**Figure 7:**
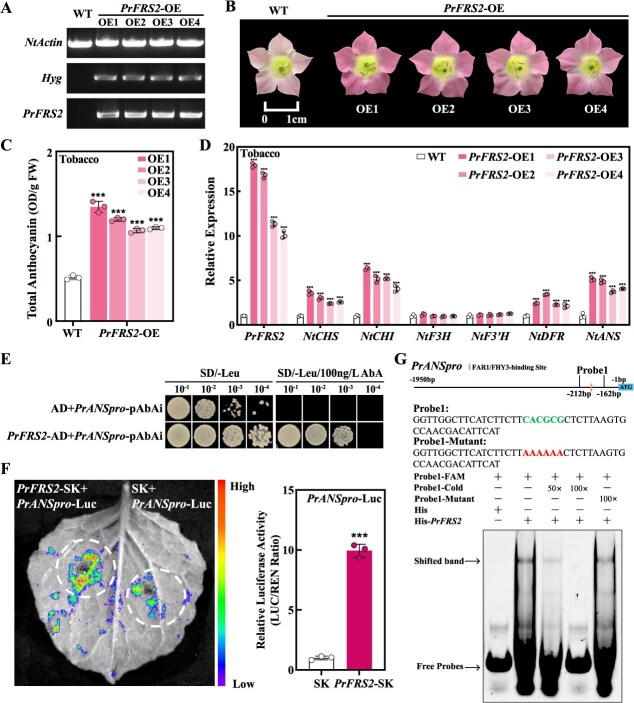
PrFRS2 directly induces anthocyanin accumulation by binding to the FBS motif in the *PrANS* promoter. (A) PCR-positive validation of WT and four *PrFRS2*-OE tobacco lines. *NtActin*: internal reference gene; *Hyg*: hygromycin resistance gene. (B) Flower phenotypes of WT and four *PrFRS2*-OE tobacco lines. (C) Total anthocyanin content in WT and four *PrFRS2*-OE tobacco lines. (D) RT-qPCR analysis of *PrFRS2* and anthocyanin structural genes (*NtCHS*, *NtCHI*, *NtF3H*, *NtF3'H*, *NtDFR*, and *NtANS*) in WT and four *PrFRS2*-OE tobacco lines. (E) PrFRS2 binds to the *PrANS* promoter in the Y1H system. Yeast cells harboring *PrFRS2*-AD and *PrANSpro*-pAbAi vectors were cultured on SD/−Leu medium with 100 ng/l AbA. The AD + *PrANSpro*-pAbAi combination served as the negative control. (F) A luminescence image (left) and relative luciferase activity (right) from Dual-luc reporter assays demonstrated that PrFRS2 activates the *PrANS* promoter. *PrFRS2*-SK and *PrANSpro*-Luc vectors were co-infiltrated into *N. benthamiana* leaves, with the SK + *PrANSpro*-Luc combination used as a control. (G) EMSA showed that PrFRS2 directly binds to the FBS motif in the *PrANS* promoter. Probe 1 (−212 to −162 bp) from *PrANSpro* contains one FBS motif (CACGCG). −FAM: FAM-labeled probes; −Cold: unlabeled competitor probes; −Mutant: unlabeled mutant probes; His: negative control protein; His-*PrFRS2*: PrFRS2 protein. + and – indicate the presence or absence of probes or proteins. 50× and 100× denote molar excesses of unlabeled probes. Data represent the mean ± SD of three biological replicates, with asterisks indicating significant differences determined by a two-sided Student’s *t*-test (^***^P < 0.001).

Although we have confirmed that PrFRS2 activates anthocyanin synthesis by regulating *PrMYB75a*, subsequent transgenic experiments revealed a direct regulatory role for *PrFRS2*. We stably transformed *PrFRS2* into tobacco plants, obtaining four transgenic lines (Fig. 7A). These *PrFRS2-*overexpressing (*PrFRS2-*OE) lines exhibited deeper red corollas and higher anthocyanin levels than WT plants (Fig. 7B and C). All transgenic lines showed increased expression of *PrFRS2*, accompanied by significant upregulation of *NtCHS*, *NtCHI*, *NtDFR*, and *NtANS* (Fig. 7D). Further analysis revealed that the FBS motif exists only in *PrANSpro* (Fig. S6). Y1H and Dual-luc reporter assays verified that PrFRS2 could bind to *PrANSpro* and enhance its activity (Fig. 7E and F). EMSA results indicated that PrFRS2 directly bound to the FBS motif (CACGCG) in *PrANSpro* (Fig. 7G). In summary, PrFRS2 functions as an activator, regulating anthocyanin accumulation by binding to *PrANSpro* and *PrMYB75apro* in the purple petals of FTP.

## Discussion

Plants commonly evolve a diversity of flower colors in response to environmental pressures and pollinator selection [47, 48]. FTP, a distinct cultivar group from CTP that originated genetically from the white-flowered wild *P. rockii*, displays various petal background colors besides white, while retaining an obvious basal flare in each flower [4, 6]. However, the regulatory mechanisms underlying the variation in petal background color in FTP remain uncharacterized. By using contrasting white- and purple-flowered cultivars, we investigate the transcriptional regulation mechanisms that control the development of petal background color in FTP.

### PrFRS2-PrMYB75a module influences petal background coloration in FTP

Although the structural genes in the ABP are well characterized in plants, including tree peony [16, 19, 49], their regulatory mechanisms require further investigation. In this study, we identified the R2R3-MYB TF PrMYB75a, which activates *PrCHS1* and *PrANS* by directly binding to MBS motifs in their promoters. This regulatory mechanism aligns with reports of R2R3-MYB TFs in tree peonies and other plants [11, 12, 24, 30]. Crucially, sequence variations in key genes are known to drive flower color diversity in lotus (*Nelumbo nucifera*), snapdragon (*Antirrhinum majus*), and violet (*Saintpaulia ionantha*) [50–52]. We detected no coding sequence (CDS) differences in *PrMYB75a* (Fig. S7) or any promoter variations in *PrCHS1* and *PrANS* between white and purple FTP cultivars (Figs S8 and S9). This finding rules out DNA sequence variation in these candidate genes as the cause of background color divergence in FTP. Instead, differential expression profiles of *PrMYB75a* between cultivars are the primary determinant of FTP petal background coloration.

The expression of *MYB* genes is often controlled by upstream TFs [25, 37]. We identified PrFRS2 as an upstream regulator of *PrMYB75a*, whose expression pattern correlates with the trend in petal anthocyanin accumulation. Functional verification proved that PrFRS2 is a positive regulator of anthocyanin synthesis. Phylogenetic analysis showed that PrFRS2 belongs to the SG1 FRS family, which is known for its involvement in plant growth and environmental stress, such as FHY3 and FAR1 in *A. thaliana* [38, 53]. Although FRS TFs have been linked to pigment accumulation in some species, their regulatory mechanisms remain unclear [40–43, 45]. We discovered that PrFRS2 promotes anthocyanin synthesis in FTP through two pathways. One pathway is by activating *PrMYB75a* to upregulate anthocyanin structural genes indirectly; the other is by directly binding to and activating *PrANS*. TFs regulating both *MYB* and structural genes have been identified in other plants, including FaRAV1 in strawberry, PuERF27 in pear, and PpNAP4 in peach (*Prunus persica*) [54–56]. Here, we identified a novel regulatory module in FTP, as PrFRS2 co-regulates *PrMYB75a* and structural genes to facilitate petal background coloration. These findings provide new perspectives for a comprehensive investigation of the plant pigment regulatory network.

In most plants, FRS TFs typically respond to various environmental signals and show distinct expression patterns [44, 57]. We found no variation in the CDS region of *PrFRS2* among FTP cultivars (Fig. S10), suggesting its function is likely modulated by upstream factors such as environmental signals. Supporting this, high-temperature signals activate *VvFHY3* in grapevine, thereby enhancing anthocyanin accumulation in fruit skin [41]. Similarly, previous studies have indicated that variations in climatic conditions during cultivation significantly influence flower coloration in tree peonies. For example, reduced light intensity causes the background color of petals to fade in cultivars ‘Taiyo’ and ‘Higuishi’ [36, 58]. Therefore, investigating how environmental signals regulate anthocyanin synthesis through the PrFRS2-PrMYB75a module in further study is essential for both theoretical understanding and breeding applications.

### Distinct mechanisms regulating petal background and basal flare coloration

Flowering plants usually develop complex pigmentation patterns (variegated flowers) to enhance pollinator attraction [59, 60]. In FTP petals, such patterns include basal flares (flower spots) formed by uneven anthocyanin accumulation in different regions of the petals [11, 61]. Previous studies on FTP have mainly focused on flare formation [11–13], while background coloration received less attention. Our study found that anthocyanin compounds contributing to both background coloration and flare formation are similar in FTP petals [14, 15]. However, the background color appears later than the flare, suggesting that these two coloration processes are relatively independent of each other.

Previous research in FTP has established that basal flare formation relies on the spatiotemporal expression of *MYB*s (*PsMYB12* and *PrMYB5* are only highly expressed in flares) [11, 12] or epigenetic modifications of promoters (PrMYBa1/PrMYBa3 specifically binds to hypomethylated promoters of *PrF3H* and *PrANS* in flares) [13]. In contrast, we demonstrated that *PrMYB75a*, when expressed in the petals, disregards spatial constraints. It is highly expressed in both flared and nonflared regions of purple petals, but not in whole petals of the white cultivar (Fig. S11), indicating a specific role in background coloration. Among spotted flowers, different MYB TFs have been reported to independently influence background and spot coloration [62–64]. For instance, *CgMYB6* controls background coloration in *Clarkia unguiculata*, while three other genes (*CgMYB1*, *CgMYB11*, and *CgMYB12*) are responsible for spot formation [65]. These findings collectively suggest that the independent regulation of background and flare pigmentation in petals is conserved across variegated plants, warranting further exploration into its mechanistic basis and potential biological significance.

**Figure 8:**
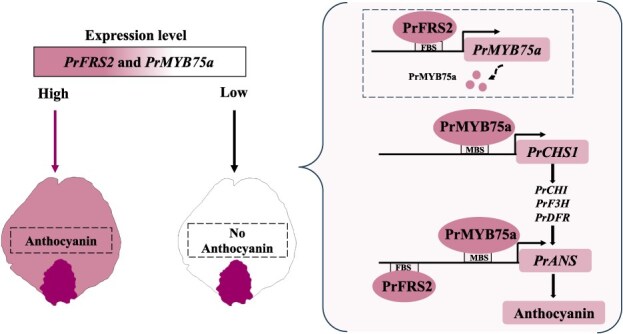
A proposed regulatory mechanism of the PrFRS2-PrMYB75a module controls the petal background coloration in *P. rockii*. In purple petals, PrMYB75a positively regulates anthocyanin synthesis by directly binding to MBS motifs in the promoters of *PrCHS1* and *PrANS*. Moreover, the FRS TF PrFRS2 acts upstream, binding to FBS motifs in both *PrMYB75a* and *PrANS* promoters to enhance anthocyanin production. In white petals, low expression levels of *PrFRS2* and *PrMYB75a* decrease the transcription of the anthocyanin structural gene, resulting in the absence of pigment deposition in the background region.

In conclusion, we have identified a PrFRS2-PrMYB75a model that regulates petal background coloration in FTP. PrFRS2 promotes anthocyanin production and petal coloration by activating *PrMYB75a* to indirectly upregulate *PrCHS1* and *PrANS*, or directly activating *PrANS* by binding to its promoter (Fig. 8). Additionally, background and basal flare coloration develop independently, with *PrMYB75a* governing uniform background coloration, while different *PrMYBs* regulate flare formation. Collectively, our study advances the understanding of floral color evolution and complex patterning mechanisms in ornamentals, providing molecular targets for precision breeding in tree peonies.

## Materials and methods

### Plant material and growth conditions

*Paeonia rockii* cultivars ‘Jing Hong’ (JH) and ‘Jing Yu Dan’ (JYD) were cultivated in the Guose Tree Peony Garden (Beijing, China; 40°45′N, 115°97′E). Petals were collected at five developmental stages. Stage 1 (S1): petals are green with no flare; Stage 2 (S2): petals have a purple flare, but the petal color remains green; Stage 3 (S3): petals are slightly colored; Stage 4 (S4): flowers begin to bloom; and Stage 5 (S5): flowers are fully bloomed. Basal flare and nonflare (petals excluding the basal flare) regions were sampled separately, immediately frozen in liquid nitrogen, and stored at −80°C for further analysis. *Nicotiana tabacum* ‘NC89’ and *A. thaliana* ecotype Col-0 were used for stable genetic transformation experiments. *Nicotiana benthamiana* was used for subcellular localization and dual-luciferase reporter assays. All tobacco and *Arabidopsis* plants were grown under a 16-/8-h (day/night) photoperiod at 22°C in an artificial climate chamber.

### Total anthocyanin measurement

Approximately 100–200 mg (Fresh weight, FW) of plant materials were ground into a powder in liquid nitrogen and subsequently mixed with 1 ml of extraction solution (methanol/ultrapure water/formic acid/trifluoroacetic acid = 70:27:2:1, v/v/v/v) at 4°C for 24 h. The supernatant was filtered through a 0.22-μm membrane after centrifugation. The UV spectrophotometer was employed to measure the absorbance, and the OD values were used to represent the relative anthocyanin content according to previous research [66, 67]. Anthocyanin content in the FTP petals and tobacco flowers was calculated by measuring absorbance at 525 nm (OD_525_). Anthocyanin content in *Arabidopsis* was calculated by subtracting OD_657_ from OD_525_.

### High-performance liquid chromatography-ion trap mass

HPLC-MS analysis was conducted on petals (excluding the basal flare) from JH and JYD cultivars at S4. The analytical platform consisted of an Agilent 1100 series HPLC-MS Trap VL instrument (Agilent Technologies, Santa Clara, CA) equipped with photodiode array detection. Separation was achieved using a Tosoh ODS-80Ts QA reversed-phase column (5 μm, 4.6 × 250 mm) protected by an ANPEL C18 guard cartridge (Shanghai ANPEL). The mobile phase comprised two components: solvent A (an aqueous phase containing 97.9% ultrapure water, 2% formic acid, and 0.1% trifluoroacetic acid) and solvent B (an organic phase comprising 62.9% acetonitrile, 35% formic acid, 2% methanol, and 0.1% trifluoroacetic acid). A linear binary gradient elution program was implemented for compound separation, with anthocyanin detection optimized at 525 nm. Cyanin chloride (Sigma-Aldrich, MO, USA) served as the reference standard for quantitative analysis. The mass spectral data were compared with those from tree peony cultivars in a previous study [14].

### Total RNA extraction and gene expression analysis

Total RNA was extracted from FTP petals, *Arabidopsis* plants, and tobacco flowers. cDNA synthesis was performed as the methods previously described [68]. Primers were designed using Primer 5.0, and all sequences are listed in Table S9. qPCR was performed using the SYBR Green Premix *Pro Taq* HS qPCR kit II (Accurate Biotechnology, Changsha, China) with three biological replicates. The experiments were carried out on a qTOWER 2.2 Real-Time PCR System (Analytik Jena, Germany). The relative expression levels of target genes were calculated using the formula 2^−ΔΔCt^ method. *Paeonia rockii Ubiquitin* (*PrUBQ*), *Arabidopsis AtPP2A*, and tobacco *NtActin* were used as the internal reference genes.

### Cloning of the full-length coding sequences of genes

The cDNA libraries from JH and JYD were used to clone the full-length coding sequences (CDSs) of *PrMYB75a* and *PrFRS2* using *ApexHF* HS DNA Polymerase CL (Accurate Biotechnology, Changsha, China). Primers were designed with SnapGene software (version 8.0), and all sequences are listed in Table S9.

### Phylogenetic analysis and multiple sequence alignment

MEGA software (version 4.0) was used to generate neighbor-joining phylogenetic trees. These trees featured pairwise deletions to account for missing data, uniform rates across sites, and the p-distance method for amino acid substitutions. Clade support was tested using 1000 bootstrap replicates. Multiple sequence alignment of PrMYB75a and its highly homologous MYBs was carried out using DNAMAN (version 9.0). All MYB TFs and FRS TFs from *Arabidopsis* and other plant species were downloaded from PlantTFDB (https://planttfdb.gao-lab.org/) and NCBI (https://www.ncbi.nlm.nih.gov/).

### Subcellular localization

The CDSs of *PrMYB75a* and *PrFRS2*, lacking the stop codons, were inserted into the pNC-Amp-GFP-N vector using the Ready-to-Use Seamless Cloning Kit (Sangon Biotech, Shanghai, China) (Fig. S12A). The empty GFP vector was used as a negative control. A nuclear localization signal (NLS) marker fused to the red fluorescent protein mCherry (NLS-mCherry) was used to indicate the nuclear subcellular localization. *Agrobacterium* strains GV3101 (pSoup-p19) harboring the recombinant plasmid, empty vector, and NLS-mCherry were grown to an OD_600_ of 0.6 and then infiltrated into the leaves of 4-week-old *N. benthamiana* plants following the described protocol [69]. After 48 h, the GFP and mCherry fluorescence signals were observed using a laser scanning confocal microscope (Olympus Corporation, Japan). GFP fluorescence was excited with a 488-nm laser and collected within the 495- to 545-nm range; mCherry fluorescence was excited with a 552-nm laser and collected within the 600- to 650-nm range. All primers are listed in Table S9.

### *Arabidopsis* transformation

The CDS of *PrMYB75a* was inserted into the pNC-Cam1304-*35S* vector (Fig. S12B). The recombinant vectors were introduced into *the Agrobacterium* strain GV3101 and then transformed into *Arabidopsis* Col-0 via the floral-dip method, as described by Clough and Bent [70]. To identify positive transgenic lines, PCR was performed to detect the specific fragments of the *Hyg* and *PrMYB75a* in the T_3_ plant. The phenotypes of positive lines were then observed and photographed using a stereo microscope (Leica, Germany). Anthocyanin content and gene expression in *PrMYB75a*-OE and WT *Arabidopsis* plants were measured, and all primers are listed in Table S9.

### Tobacco transformation

The CDSs of *PrMYB75a* and *PrFRS2* were introduced into the pNC-Cam1304-35S vector (Fig. S12B) and transformed into *the Agrobacterium* strain GV3101. Transgenic tobacco plants were generated using the leaf disc method in *N. tabacum* ‘NC89’, as previously described [71]. PCR analysis was performed to detect specific fragments of *Hyg*, *PrMYB75a*, and *PrFRS2* in positive transgenic lines. Flower morphology was observed and photographed with a Sony camera (Tokyo, Japan). Anthocyanin content and gene expression in *PrMYB75a*-OE, *PrFRS2*-OE, and WT tobacco flowers were measured, and all primers are listed in Table S9.

### VIGS in tree peony petals

Specific CDS fragments of *PrMYB75a* and *PrFRS2* were cloned into the pTRV2 vector (Fig. S12C). The empty vectors (pTRV1 and pTRV2) and recombinant vectors were introduced into *Agrobacterium* strain GV3101. *Agrobacterium* strain GV3101 containing pTRV1 and recombinant pTRV2 vectors was resuspended to an OD_600_ of 1.0 in infiltration buffer (10 mM MgCl_2_, 200 μM acetosyringone, 10 mM MES, pH 5.6) and then mixed at a 1:1 ratio. A mixture of pTRV1 and empty pTRV2 served as the control treatment (EV). JH buds with 5-cm stalks at S2 were subjected to vacuum infiltration at 0.8 kg/cm^2^ for 5 min twice. The stalks were cultivated in the dark with water at 8°C for 1 day, then maintained at 23°C under 16 h/8 h (day/night) photoperiod and 60% humidity for 5 days. Specific fragments from pTRV1, pTRV2, and target genes (*PrMYB75a* and *PrFRS2*) were detected in silenced lines using semiquantitative PCR (SQ-PCR). Petal phenotypes were observed and photographed using a Sony camera (Tokyo, Japan). Anthocyanin content and gene expression in *PrMYB75a*-SL, *PrFRS2*-SL, EV, and WT petals were analyzed, and all primers are listed in Table S9.

### Promoter sequence isolation and analysis

Promoter sequences of key genes were cloned from the genomic DNA of JH or JYD petals using *ApexHF* HS DNA Polymerase CL (Accurate Biotechnology, Changsha, China). Specific primers were designed based on the JH genome data (unpublished). Cis-elements in the promoter regions were analyzed using the PlantCARE website (http://bioinformatics.psb.ugent.be/webtools/plantcare/html).

### Yeast one-hybrid assay

The CDSs of *PrMYB75a* and *PrFRS2* were inserted into pNC-GADT7 bait vectors, while promoter regions of *PrCHS1*, *PrCHS1pro*-1 (−2000 to −525 bp) and *PrCHS1pro*-*2* (−2000 to −1218 bp), *PrCHI*, *PrDFR*, *PrF3H*, *PrANS*, and *PrMYB75a* were inserted into pNC-AbAi prey vectors (Fig. S12D). Then the Y1H assay was performed as described previously [72]. Bait constructs were combined with the Y1H Gold strain using the Classic Yeast Competent Cell Preparation and Transformation Kit (Coolaber Science & Technology, Beijing, China). Prey constructs were subsequently transformed into Y1H yeast cells with prey constructs. A single colony was picked and diluted 1-, 10-, 100-, or 1000-fold before being inoculated into SD/−Leu medium supplemented with appropriate concentrations of Aureobasidin A (AbA) (Coolaber Science & Technology, Beijing, China). The empty vector (AD) was used as the negative control, and all primers used are listed in Table S9.

### Dual-luciferase reporter assay

The CDSs of *PrMYB75a* and *PrFRS2* were inserted into pNC-Green62-SK effector vectors, and the promoters of *PrCHS1*, *PrCHS1pro*-1 (−2000 to −525 bp) and *PrCHS1pro*-*2* (−2000 to −1218 bp), *PrCHI*, *PrDFR*, *PrF3H*, *PrANS*, and *PrMYB75a* were inserted into pNC-Green62-Luc reporter vectors (Fig. S12E). All effector and reporter constructs were introduced into the *Agrobacterium* strain GV3101 (pSoup-p19), which was then treated according to the subcellular localization assay described above. *Agrobacterium-*mediated recombinant effector and reporter constructs were co-infiltrated into the leaves of 4-week-old *N. benthamiana* plants at a 1:1 ratio. After 48 h, the firefly luciferase (Luc) activity was photographed after spraying with 2.5 mM D-luciferin potassium (Solarbio Science & Technology, Beijing, China) using the NightOwl LB 983 Plant Imaging System (Berthold Technologies, Germany). Subsequently, the activities of Luc and Renilla luciferase (Ren) were measured using the Dual Luciferase Reporter Gene Assay Kit (Yeasen Biotechnology, Shanghai, China) and a BioTek Synergy HTX (Agilent, USA). All primers are listed in Table S9.

### Electrophoretic mobility shift assay

The CDSs of *PrMYB75a* and *PrFRS2* were introduced into the pNC-ET28 vector, and the recombinant vectors were transformed into *Escherichia coli* strain BL21(DE3) for protein expression in prokaryotic systems (Fig. S12F). The induced proteins were purified using an AKTA Pure Chromatography System (Cytiva, Sweden). Both unlabeled and 5(6)-carboxyfluorescein (FAM)-labeled oligonucleotide probes were synthesized by Sangon Biotech (Shanghai, China). The purified proteins and probes were used in the EMSA experiments. Unlabeled probes served as cold competitors, while mutant probes were used as mutant competitors. EMSA experiments were conducted as described in a previous study [73]. DNA–protein complexes were separated using 6% native polyacrylamide gels. The gels were imaged using a ChemiDoc MP Imaging System (Bio-Rad, USA). All primers are listed in Table S9.

### Y1H screening

To generate the bait construct, the promoter of *PrMYB75a* was inserted into the pNC-AbAi vector (Fig. S12D). Then the bait construct was integrated into the chromosome of the Y1H Gold strain. The TF library from JH petals (excluding basal flare) was constructed by OE Biotech (Shanghai, China). The library plasmids were transformed into the Y1H strain containing the bait construct and screened on the selective medium SD/−Leu supplemented with 200 ng/l AbA. Yeast clones that survived the selection were then sequenced. Sequencing-confirmed yeast clones were subjected to point-to-point validation to confirm that the TFs could bind to the *PrMYB75a* promoter. All primers are listed in Table S9.

## Supplementary Material

Web_Material_uhaf288

## Data Availability

The transcriptome databases used in this study are available at the China National Gene Bank under accession number PRJCA030598.
