# Supplementary material for: The PrFRS2-PrMYB75a module regulates petal coloration in flare tree peony (Paeonia rockii)
Source: Hortic Res. 2025 Oct 16;13(2):uhaf288. doi: 10.1093/hr/uhaf288 (PMC12903464; doi:10.1093/hr/uhaf288)
Supplement: Web_Material_uhaf288 [file web_material_uhaf288.zip › Supplementary figures.docx]

**Figure S1. The proportion of different anthocyanin components in JH petals (excluding the basal flare) at S4.** Data were obtained by HPLC-MS analysis at 525nm, using Cyanin chloride as the reference standard. Cy3G: cyanidin 3-*O*-glucoside; Cy3G5G: cyanidin 3,5-di-*O*-glucoside; Pn3G5G: peonidin 3,5-di-*O*-glucoside; Pn3G: peonidin 3-*O*-glucoside; Pg3G5G: pelargonidin 3,5-di-*O*-glucoside.


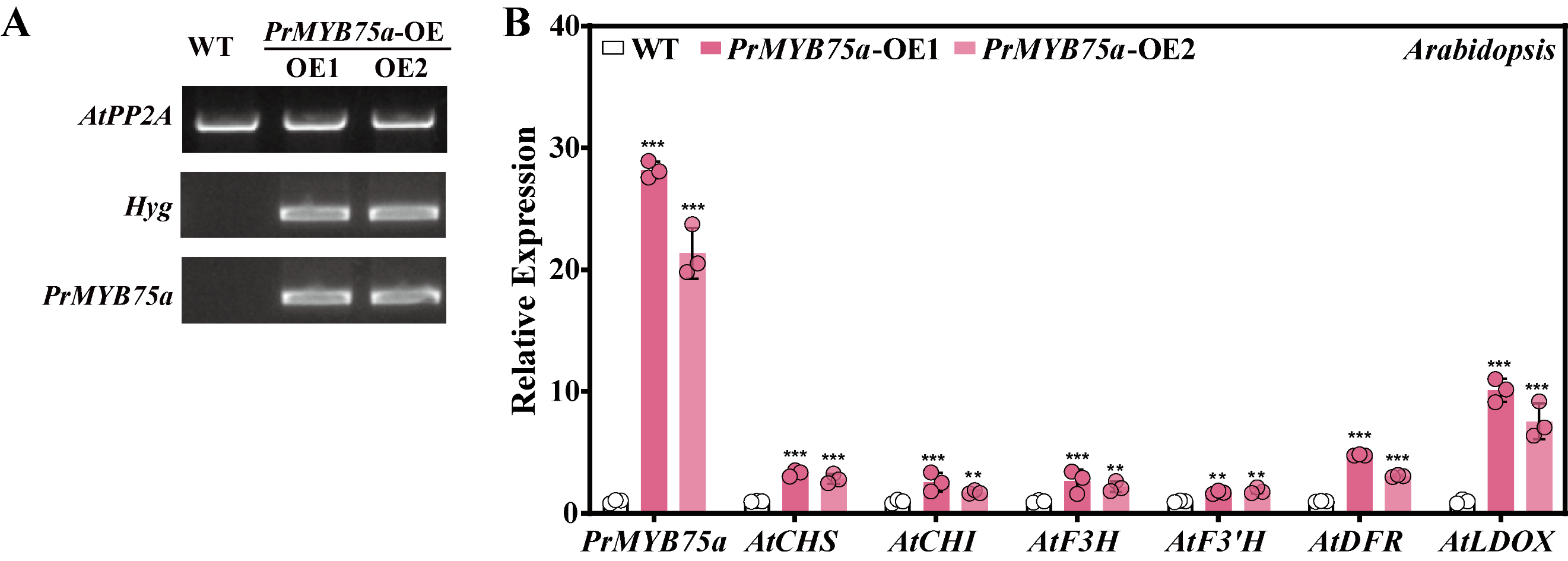


**Figure S2. Analysis of WT and** **two *PrMYB75a*-OE *Arabidopsis* plants. (A)** PCR-positive verification. *AtPP2A*: internal reference gene; *Hyg*: hygromycin resistance gene. **(B)** Relative expression levels of *PrMYB75a* and anthocyanin biosynthesis genes (*AtCHS*, *AtCHI*, *AtF3H*, *AtF3'H*, *AtDFR*, and *AtLDOX*) by RT-qPCR assay. Data represent the mean ± standard deviation (SD) of three biological replicates. Asterisks indicate significant differences determined by a two-sided Student’s *t*-test (***P*<0.01, ****P*<0.001).


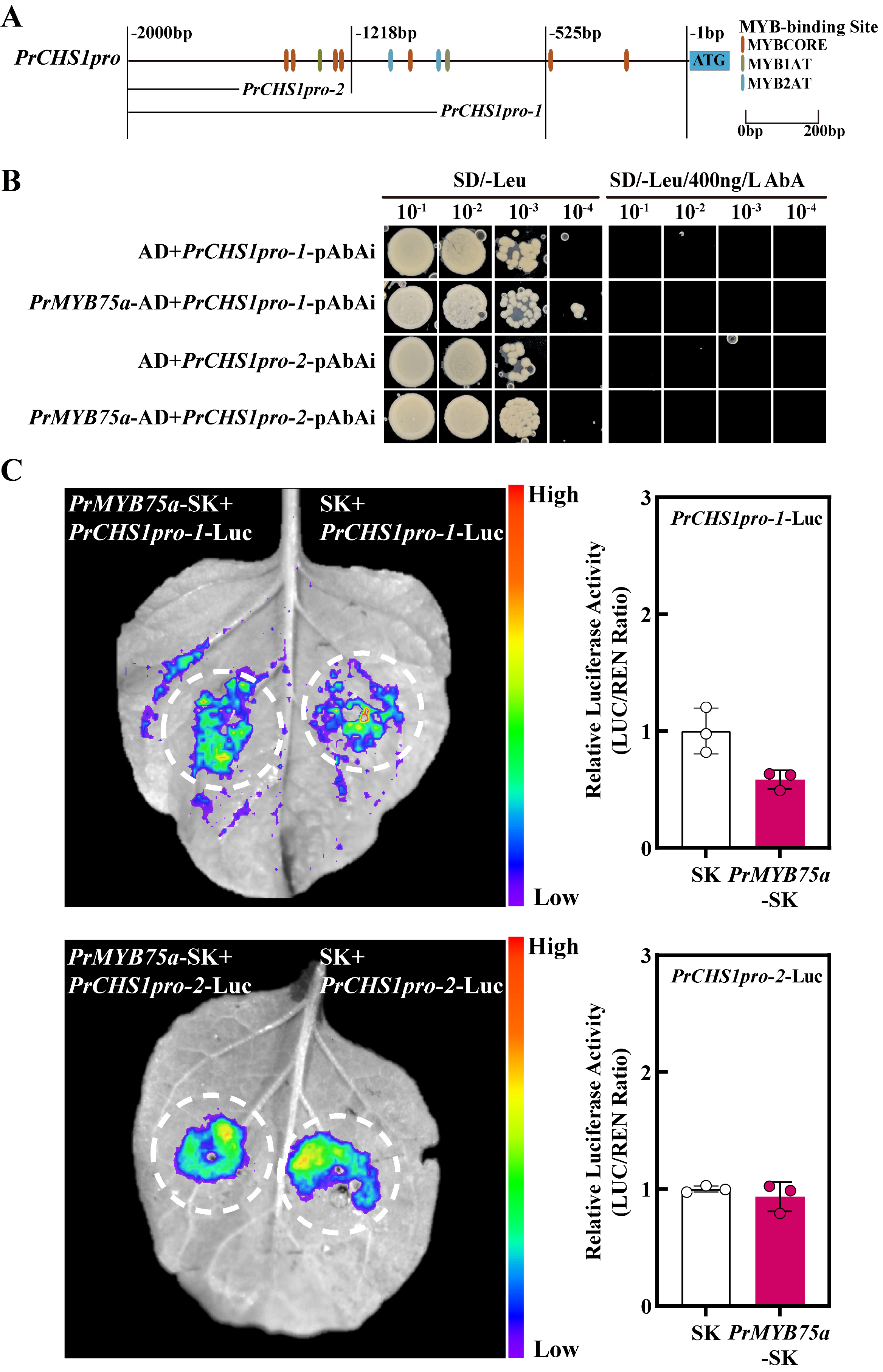


**Figure S3. PrMYB75a cannot bind to the two shortened fragments of the *PrCHS1* promoter*.* (A)** Two shortened fragments of *PrCHS1pro*. Different MBS motifs (MYBCORE, MYB1AT, MYB2AT, and MYB2CONSENSUSAT) are highlighted with colored rectangles. *PrCHS1pro-1*: -2000bp to -525bp region of *PrCHS1pro*, with nine MBS motifs; *PrCHS1pro-2*: -2000bp to -1218bp region of *PrCHS1pro*, with five MBS motifs. **(B)** The Y1H assay showed that PrMYB75a cannot bind to the *PrCHS1pro-1*/*2*. Yeast cells containing *PrMYB75a*-AD and *PrCHS1pro-1/2*-pAbAi constructs were cultured on SD/-Leu medium with 100ng/L AbA. AD: empty AD vector served as the control. **(C)** Dual-luc reporter assay showed that PrMYB75a cannot promote the activity of *PrCHS1pro-1/2.* Effector *PrMYB75a*-SK and the reporter constructs (*PrCHS1pro*-*1/2*-Luc) were co-infiltrated into *N. benthamiana* leaves. The SK+ *PrCHS1pro*-*1/2*-Luc combinations were used as the control; Representative images show Luc luminescence in leaves (left), LUC/REN ratio reflects relative luciferase activity(right), with the control set to 1. Data represent the mean ± SD of three biological replicates, with asterisks indicating significant differences determined by a two-sided Student’s *t*-test (****P*<0.001).

**Figure S4. The promoter sequences of *PrMYB75a* were cloned from the JH and JYD petals.**


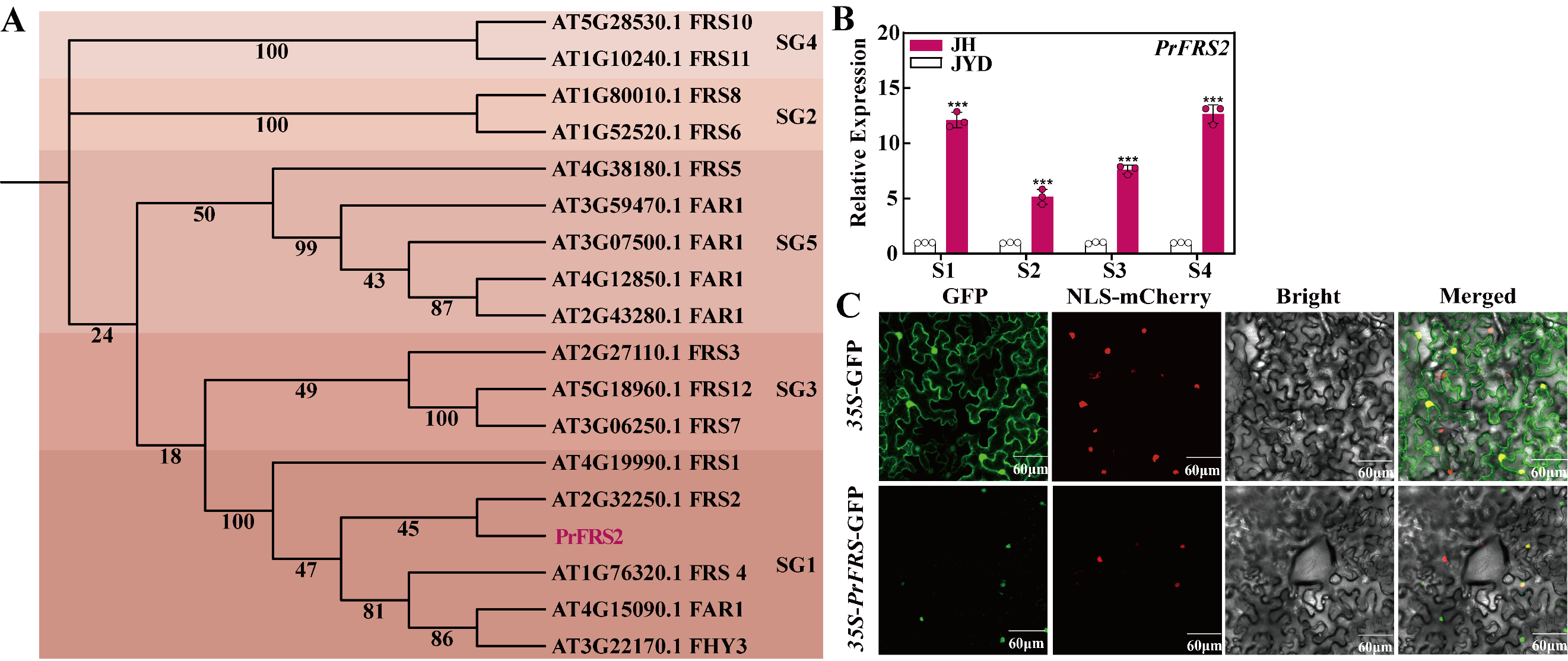


**Figure S5. Phylogenetic, gene expression, and subcellular localization analyses of PrFRS2. (A)** The phylogenetic tree was constructed using the amino acid sequences of PrFRS2 and 17 *Arabidopsis* FRS TFs. This analysis was performed using MEGA4 with a p-distance model and 1000 bootstrap replicates. PrFRS2 is highlighted in red. Accession numbers of TFs are listed in **Table S8**. **(B)** Relative expression levels of *PrFRS2* in JH and JYD petals (excluding the basal flare) at S1-S4 by RT-qPCR assay. Data represent the mean ± SD of three biological replicates, with asterisks indicating significant differences determined by a two-sided Student’s *t*-test (****P*<0.001). **(C)** Subcellular localization of PrFRS2 in the epidermal cells of *N. benthamiana* leaves. The *35S*-*PrFRS2*-GFP vector was co-infiltrated with the nuclear marker mCherry protein. The empty GFP vector (*35S*-GFP) with mCherry protein served as a control. GFP: GFP channel; NLS-mCherry: nuclear localization signal; Bright: light microscopy image; Merged: merged image of the GFP, NLS-mCherry, and Bright channels.


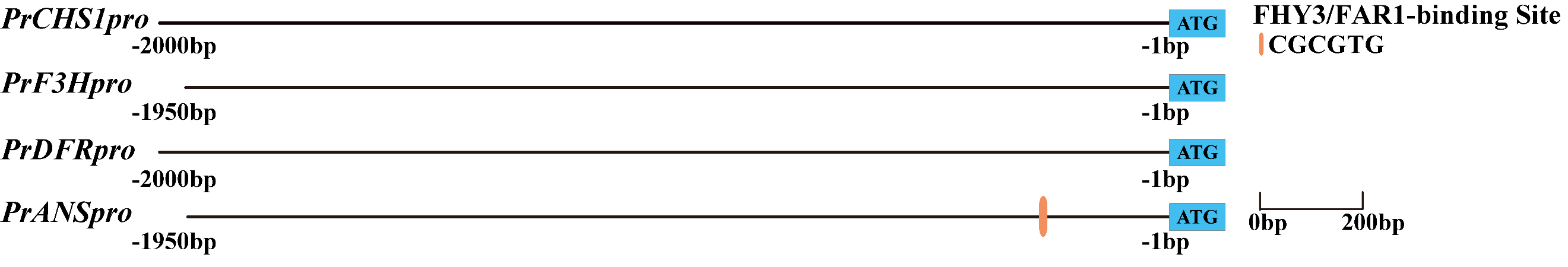


**Figure S6. Potential FHY3/FAR1-binding site (FBS) in the promoters of *PrCHS1*, *PrF3H*, *PrDFR*, and *PrANS*.**

**Figure S7. The CDS regions of *PrMYB75a* were cloned from the JH and JYD petals (excluding basal flare).**

**Figure S8. The promoter sequences of *PrCHS1* were cloned from the JH and JYD petals.**

**Figure S9. The promoter sequences of *PrANS* were cloned from the JH and JYD petals.**

**Figure S10. The CDS regions of *PrFRS2* were cloned from the JH and JYD petals (excluding basal flare).**


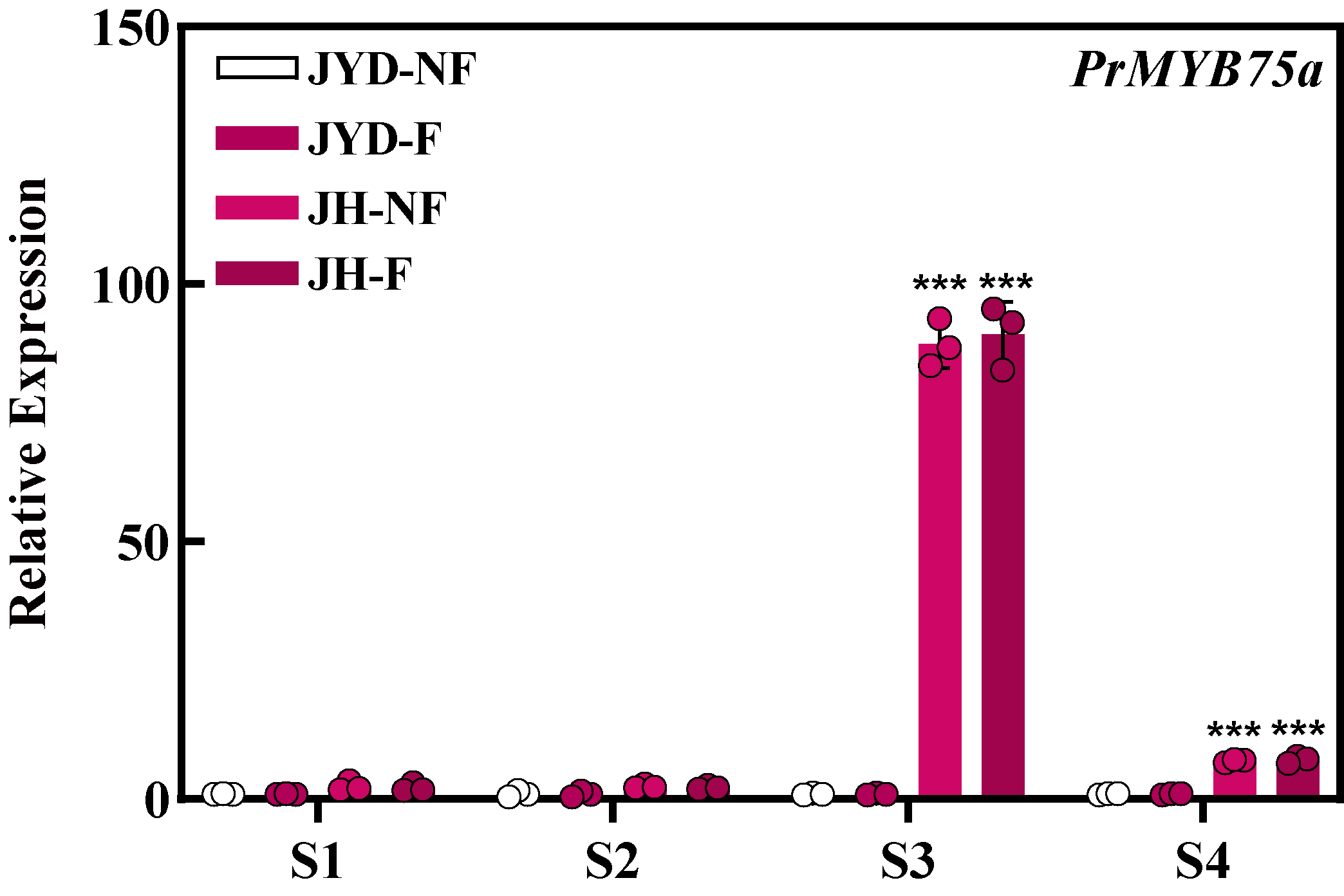


**Figure S11. Relative expression profile of *PrMYB75a* in non-flared and flared regions in JH and JYD petals at S1-S4 by RT-qPCR.** -NF: non-flared regions (excluding the basal flare) in petals; -F: flared regions in petals. Data represent the mean ± SD of three biological replicates, with asterisks indicating significant differences determined by a two-sided Student’s *t*-test (****P*<0.001).

**
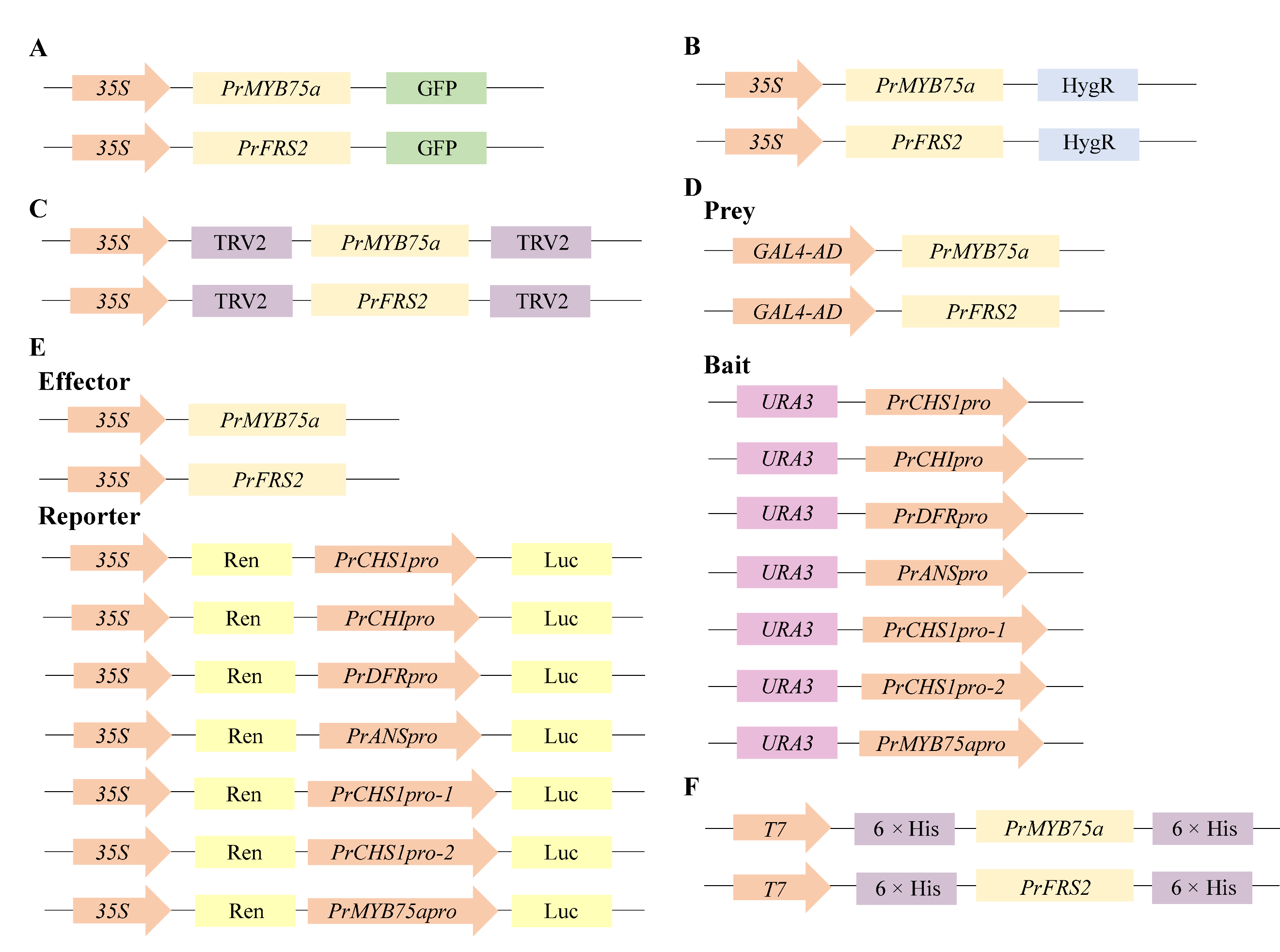
**

**Figure S12. Structure diagrams of the recombinant vectors used in this study. (A)** The *PrMYB75a* and *PrFRS2* CDSs without the stop codons were inserted into pNC-Amp-GFP-N vectors driven by the *35S* promoter to generate *35S*-*PrMYB75a*-GFP and *35S*-*PrFRS2*-GFP vectors. **(B)** The *PrMYB75a* and *PrFRS2* CDSs were inserted into pNC-Cam1304-*35S* vectors driven by the *35S* promoter to generate *35S*-*PrMYB75a*-1304 and *35S*-*PrFRS2*-1304 vectors. **(C)** The 200 bp fragments from non-conservative CDS of *PrMYB75a* and *PrFRS2* were inserted into pTRV2 vectors to generate *PrMYB75a*-TRV2 and *PrFRS2*-TRV2 vectors. **(D)** The *PrMYB75a* and *PrFRS2* CDSs were inserted into pNC-GADT7 prey vectors driven by the *GAL4-AD* promoter to generate AD-*PrMYB75a* and AD-*PrFRS2* vectors. The *PrCHS1pro*, *PrCHS1pro-1* (-2000 bp to -525 bp) and *PrCHS1pro-2* (-2000 bp to -1218 bp), *PrCHIpro*, *PrF3Hpro*, *PrDFRpro*, *PrANSpro*, and *PrMYB75apro* were inserted into pNC-pAbAi bait vectors to generate *PrCHS1pro-*pAbAi, *PrCHS1pro-1-*pAbAi, *PrCHS1pro-2-*pAbAi, *PrCHIpro-*pAbAi, *PrF3Hpro-*pAbAi, *PrDFRpro-*pAbAi, *PrANSpro-*pAbAi, and *PrMYB75apro-*pAbAi. **(E)** The *PrMYB75a* and *PrFRS2* CDSs were inserted into pNC-Green62-SK effector vectors driven by the *35S* promoter to generate *PrMYB75a*-SK and *PrFRS2*-SK vectors. The *PrCHS1pro*, *PrCHS1pro-1* (-515 bp to -2000 bp) and *PrCHS1pro-2* (-1218 bp to -2000 bp), *PrCHIpro*, *PrF3Hpro*, *PrDFRpro*, *PrANSpro*, and *PrMYB75apro* were inserted into pNC-Green62-Luc reporter vectors to generate *PrCHS1pro-*Luc, *PrCHS1pro-1-*Luc, *PrCHS1pro-2-*Luc, *PrCHIpro-*Luc, *PrF3Hpro-*Luc, *PrDFRpro-*Luc, *PrANSpro-*Luc, and *PrMYB75apro-*Luc. **(F)** The *PrMYB75a* and *PrFRS2* CDSs were inserted into pNC-ET28 vectors driven by the *T7* promoter to generate His-*PrMYB75a* and His-*PrFRS2* vectors.
